# Myeloid-Derived Suppressor Cells Induce Podocyte Injury Through Increasing Reactive Oxygen Species in Lupus Nephritis

**DOI:** 10.3389/fimmu.2018.01443

**Published:** 2018-06-25

**Authors:** Dongya Zhang, Jingjing Xu, Jing Ren, Liang Ding, Guoping Shi, Dan Li, Huan Dou, Yayi Hou

**Affiliations:** ^1^The State Key Laboratory of Pharmaceutical Biotechnology, Division of Immunology, Medical School, Nanjing University, Nanjing, China; ^2^Jiangsu Key Laboratory of Molecular Medicine, Nanjing, China

**Keywords:** lupus nephritis, myeloid-derived suppressor cells, podocytes, toll-like receptor-7, reactive oxygen species

## Abstract

The expansion of myeloid-derived suppressor cells (MDSCs) has been documented in murine models and patients with lupus nephritis (LN), but the exact role of MDSCs in this process remains largely unknown. In this study, we investigated whether MDSCs are involved in the process of podocyte injury in the development of LN. In toll-like receptor-7 (TLR-7) agonist imiquimod-induced lupus mice, we found the severe podocyte injury in glomeruli of lupus mice and significant expansion of MDSCs in spleens and kidneys of lupus mice. The function of TLR-7 activated MDSCs was enhanced including the increased generation of reactive oxygen species (ROS) *in vivo* and in *vitro*. Moreover, the ROS production of MDSCs induced podocyte injury through activating the p-38MAPK and NF-kB signaling. Furthermore, we verified that podocyte injury was indeed correlated with expansion of MDSCs and their ROS secretion in LN of pristane-induced lupus mice. These findings first indicate that the podocyte injury in LN was associated with the increased MDSCs in kidney and MDSCs may be a promising therapeutic target of LN in the future.

## Introduction

Systemic lupus erythematosus (SLE) is a typical autoimmune disease and mostly occurs in females (9:1 prevalence) around child-bearing age (1). Lupus nephritis (LN), one of the most severe manifestations of SLE, is characterized by proteinuria, hematuria, and renal failure caused by deposition of immune complexes and tightly associated with high morbidity and mortality (2–4). Although there have been great advances in treatment and improved prognosis for patients with LN for over the past 30 years, LN treatment remains challenging. Indeed, only 50–70% of patients with LN achieved remission and 10–20% of patients will progress to end-stage renal disease within 5 years of diagnosis (5). In order to find an effective treatment, the detailed pathogenic mechanism of LN is still needed to be clarified.

Myeloid-derived suppressor cells (MDSCs) are a heterogeneous collection of myeloid cells comprised myeloid precursors, immature granulocytes, mononuclear macrophages, and dendritic cells (6, 7). MDSCs are broadly characterized by CD11b^+^ Gr-1^+^ cells in mice and HLA-DR^−^CD11b^+^CD33^+^ cells in humans, which further divided into granulocytic polymorphonuclear MDSCs (G-MDSCs) and monocytic MDSCs (M-MDSCs). G-MDSCs are phenotypically and morphologically similar to neutrophils, whereas M-MDSCs are similar to monocytes (8). Increasing evidence suggests that MDSCs exert a powerful influence on the regulation of autoimmunity in autoimmune diseases. It had been reported that MDSCs induced the expansion of regulatory B cells by inducible nitric oxide synthase (iNOS) to ameliorate autoimmunity in murine model of SLE (9). MDSCs suppressed the progression of collagen-induced arthritis by inhibiting the proinflammatory immune response of CD4^+^ T cells in autoimmune arthritis (10). The frequency of MDSCs was positively correlated with the levels of serum arginase-1 (Arg-1) activity, T helper 17 (TH17) responses, and disease severity in SLE patients. In addition, MDSCs were essential for the associated renal injuries in a humanized SLE model (11). Recently, new evidence revealed that MDSCs play a crucial role in the regulation of LN. The granulocyte colony stimulating factor treatment ameliorated LN through reducing the number of G-MDSCs and M-MDSCs and promoting the preferential expansion of CD4^+^CD25^+^Foxp3^+^ Tregs in the spleen and kidney of NZB/W F1 female mice (12). Consistent with these results, in our previous study, the proportion of MDSCs in the kidney gradually increased along with the progression of SLE in MRL/lpr lupus-prone mice (13). Nevertheless, laquinimod suppressed LN in (NZB × NZW) F1 prone mice partly by inducing expansion of MDSCs and promoted a shift from proinflammatory type I monocyte/macrophages to anti-inflammatory type II monocyte/macrophages (14). These studies suggest that MDSCs may play a dual role in the development of LN. Although current evidence manifests that MDSCs are involved in LN, it is still unclear on their mechanisms in LN development.

Podocytes exert crucial influence on maintaining the normal renal function as the major component of the glomerular filtration barrier (15, 16). Podocyte injury is correlated with the generation of proteinuria, one of the major features of renal dysfunction in LN (17). Indeed, podocytes injury is common in patients with LN, as shown in a large cohort study of patients with renal-biopsy-proven LN (18). It had been reported the diverse mechanisms were involved in podocyte injury, including genetic factors, inflammation, toxic injury, and metabolic disturbances (19). Previous study revealed that the activated NLRP3 inflammasomes in podocytes from lupus-prone mice and LN patients was involved in the pathogenesis of podocyte injuries and the development of proteinuria in LN (20). Furthermore, oxidant stress-mediated aldosterone/MR-induced podocyte injury by triggering both Chop-dependent apoptosis and autophagy *via* activating ER stress (21). However, it has been unclear about the effect of MDSCs on podocyte injury in LN so far.

In the present study, we found that the significant expansion of MDSCs and severe podocyte injury in glomeruli of kidneys in toll-like receptor-7 (TLR-7) agonist imiquimod or pristane-induced lupus mice. Moreover, MDSCs generated the enhanced reactive oxygen species (ROS) *via* TLR-7 activation *in vivo* or *in vitro*, since TLR-7 is thought to trigger glomerulonephritis in experimental lupus erythematosus. Furthermore, the ROS production of MDSCs could induce podocyte injury through activating the p-38MAPK and NF-kB signaling. These results first indicate that the podocyte injury in LN was associated with the increased MDSCs in kidney and MDSCs may be a promising therapeutic target of LN in the future.

## Materials and Methods

### Mice

Female BALB/c mice (6–8 weeks old) and female C57BL/6 mice (6–8 weeks old) were obtained from Model Animal Research Center of Nanjing University (Nanjing, China) and kept under pathogen-free and housing conditions in a 12-h light and dark cycle. All experiments were conducted in accordance with institutional guidelines for animal care and used based on the Guide for the Animal Care Committee at Nanjing University. 10-week-old BALB/c mice received a single intraperitoneal injection of 0.5 ml pristane or PBS and monitored for the following 7 months. C57BL/6 mice were kept for 1 week. The skin on the right ears of the mice was treated topically, every other day, with 1.25 mg of 5% imiquimod cream for 10 weeks.

### Cell Culture

The conditionally immortalized mouse podocyte cell line, MPC5 was purchased from Shanghai Zhong Qiao Xin Zhou Biotechnology Co. (Shanghai, China). Cells were cultured at 33°C in RPMI-1640 medium (Gibco BRL, Gaithersburg, MD, USA) supplemented with 10% fetal bovine serum (FBS, Gibco BRL, Gaithersburg, MD, USA) and recombinant IFN-γ (PeproTech, USA). To induce differentiation, podocytes were reseeded and cultured at 37°C in 100 cm^2^ culture dish coated with 12 mg/ml type-I collagen (BD Bioscience, Bedford, MA, USA) and in RPMI-1640 medium supplemented with 5% FBS, deprived of IFN-γ (growth restrictive conditions) for 10–13 days.

### Histopathologic Analyses

Kidney tissues were fixed with 10% paraformaldehyde, embedded in paraffin, sectioned into 4-μm-thick slices, and stained with periodic acid–Schiff reagent.

### Immunofluorescence Staining

Frozen sections of kidneys were stained with anti-CD11b (BD Pharmingen, USA) or anti-Gr-1 (BD Pharmingen, USA) followed by treatment with horseradish peroxidase-conjugated anti-rat IgG (Dako), and were visualized using diaminobenzidine (DAB) and hematoxylin as counterstaining. Frozen sections of kidneys were treated with anti-Wilms’ tumor protein (WT-1) (Merck Millipore, Bedford, MA, USA) or anti-Nephrin (Merck Millipore, Bedford, MA, USA), followed by treated with Alexa Fluor 488-conjugated goat anti-mouse IgG (Invitrogen, Carlsbad, CA, USA). Sections were analyzed by laser scanning confocal microscope (FV3000, Olympus Corporation, Japan).

To investigate oxidative stress in the kidney tissues, DHE immunofluorescence staining was performed. Frozen, optimal cutting temperature-embedded kidney tissue was cryosectioned into 10-μm-thick sections, which were stained with 10 µmol/l dihydroethidium (DHE, Molecular Probes) solution (Invitrogen, Carlsbad, CA, USA). Images were obtained by laser scanning confocal microscope (FV3000, Olympus Corporation, Japan).

### Isolation of MDSCs From Kidney and Spleen

Kidneys were cut into small fragments and digested to single-cell suspensions with 1 mg/ml collagenase type D (Roche) and 0.1 mg/ml DNase I (Roche) in HBSS (Hanks balanced saline solution) at 37°C for 30 min, the isolated cells were layered on a 30/40/75% Percoll gradient, followed by centrifugation for 20 min at 600 *g*, and collected the cells on the 40/75% interface, containing mostly leukocytes. After washing in FACS buffer, the cells were immediately processed for FACS.

Spleens were digested to single-cell suspensions with 1 mg/ml collagenase type D (Roche) and 0.1 mg/ml DNase I (Roche) in HBSS (Hanks balanced saline solution) at 37°C for 30 min. The cells were collected and suspended in ACK for the lysis of red blood cells and then centrifuge at 1,500 rpm for 5 min. After washing, collecting cells were purified from spleen using magnetic-activated cell sorting beads (Miltenyi Biotec, Auburn, CA, USA) according to the manufacture’s protocol. The purity of cells after separation was >90%. Cells were then analyzed using flow cytometer (22).

### Generation of BM-Derived MDSCs

BM cells were isolated as described previously from mice by flushing femurs and tibiae, and then BM cells were centrifuged and resuspended in culture medium supplemented with murine IL-6 and GM-CSF (both 40 ng/ml; Miltenyi Biotec, Auburn, CA, USA) and were cultured for 4 days (23, 24). Negative immune-selection of lineage-negative bone-marrow progenitors was performed using the Myeloid-Derived Suppressor Cell Isolation Kit.

### Flow Cytometry Analysis

To detect mouse MDSC subsets, cell suspensions isolated from the spleens and kidneys were first incubated with Fc-blockeranti-CD16/32 antibody (dilution 1:20, Miltenyi Biotech) for 15 min, stained and pre-incubated with anti-CD11b-APC (1 μl/test) and anti-GR-1-PE (0.3 μl/test) for 30 min at 4°C in the dark. Cells were then washed with buffer to remove the excess stains and analyzed in a FACS (Becton Dickinson, San Diego, CA, USA). To detect TLR7 of MDSCs in pristane-treated mice, after stained with anti-CD11b-APC and anti-GR-1-PE, fixed and permeabilized by BD Fixation/Permeabilization Solution Kit (BD Pharmingen, USA), stained with anti-TLR7-FITC (Invitrogen, USA) for 30 min at 4°C.

### Apoptosis Assay

Podocyte apoptosis was measured by flow cytometry using an annexin V-FITC apoptosis detection kit (Becton Dickinson, San Diego, CA, USA). Briefly, podocytes were collected and suspended in 500 µl binding buffer, followed by staining with annexin V-FITC and propidium iodide (PI) at room temperature for 5 min in the dark. After removing the unbound annexin V-FITC and PI by centrifugation, the cells were resuspended in excess binding buffer. For each measurement, at least 10,000 cells were analyzed by FACS flow cytometer (Becton Dickinson, San Diego, CA, USA).

### ROS Detection

The oxidation-sensitive dye DCFDA (Beyotime, Shanghai, China) was used to measure ROS production. Cells were incubated at 37°C in RPMI in the presence of 2.5 µM DCFDA for 30 min. Then cells were simultaneously cultured, along with DCFDA, with 1 µg/ml LPS (Sigma-Aldrich, Louis, USA) before detected by flow cytometer (Becton Dickinson, San Diego, CA, USA) (25).

### Western Blot Analysis

Western blot analysis was performed as described previously. The podocytes, MPC5 cells were lysed in buffer containing 50 mmol/l Tris–HCl, pH 8.0, 150 mmol/l NaCl, 0.02% NaN_3_, 0.1% SDS, 100 mg/l phenylmethylsulfonyl fluoride, 1 mg/l aprotinin, and 1% Triton. Cell extract was separated by SDS-PAGE and transferred onto PVDF membranes. The membranes were blocked for 1 h in TBST (10 mmol/l Tris–HCl, pH 7.4, 150 mmol/l NaCl, 0.05% Tween-20) containing 5% bovine serum albumin, incubated with primary antibodies Nephrin (Merck Millipore, Bedford, MA, USA), WT-1 (Merck Millipore), p-p38MAPK [Cell Signaling Technology (CST), Danvers, MA, USA], p-38MAPK (CST), p-p65 (CST), p-65 (CST), and GAPDH (CST) at 4°C overnight and then incubated with secondary antibodies. Bands were visualized with enhanced chemiluminescence reaction (Millipore Corp.). GAPDH was used as the loading control. Protein bands were captured and analyzed using the Lane 1D software (Sage Creation Science Co., Beijing).

### Reverse Transcription and Real-Time Quantitative PCR Analysis

Total RNA was isolated from cells or tissues using TRizol reagent (Invitrogen) according to the manufacturer’s instructions. The condition of reversed transcription for cDNA with reverse transcription system was 42°C for 5 min, 99°C for 20 min, and 4°C for 5 min. The SYBR Green PCR Master Mix (Bio RAD) according to the manufacturer’s instructions was performed for quantitative real-time RT-PCR (qRT-PCR). The qPCR assays were carried out on a Step One Plus, and data collected with this instrument. Relative gene expression was calculated with the 2^−ΔΔCT^ formula, and results were normalized to the expression of GAPDH. All the premier sequences are shown in Table S1 in Supplementary Material.

### Statistical Analysis

All statistical calculations were performed using commercially available statistical software GraphPad Prism (GraphPad, San Diego, CA, USA). Results were expressed as mean ± SEM of three independent experiments and each experiment included triplicate sets. Data were statistically evaluated by one-way ANOVA followed by Dunnett’s test between control group and multiple dose groups. Statistical significance was defined at equal to or more than 95% confidence interval or *P* ≤ 0.05.

## Results

### Podocyte Injury in TLR-7 Agonist Imiquimod-Induced Lupus Mice

**Figure 1:**
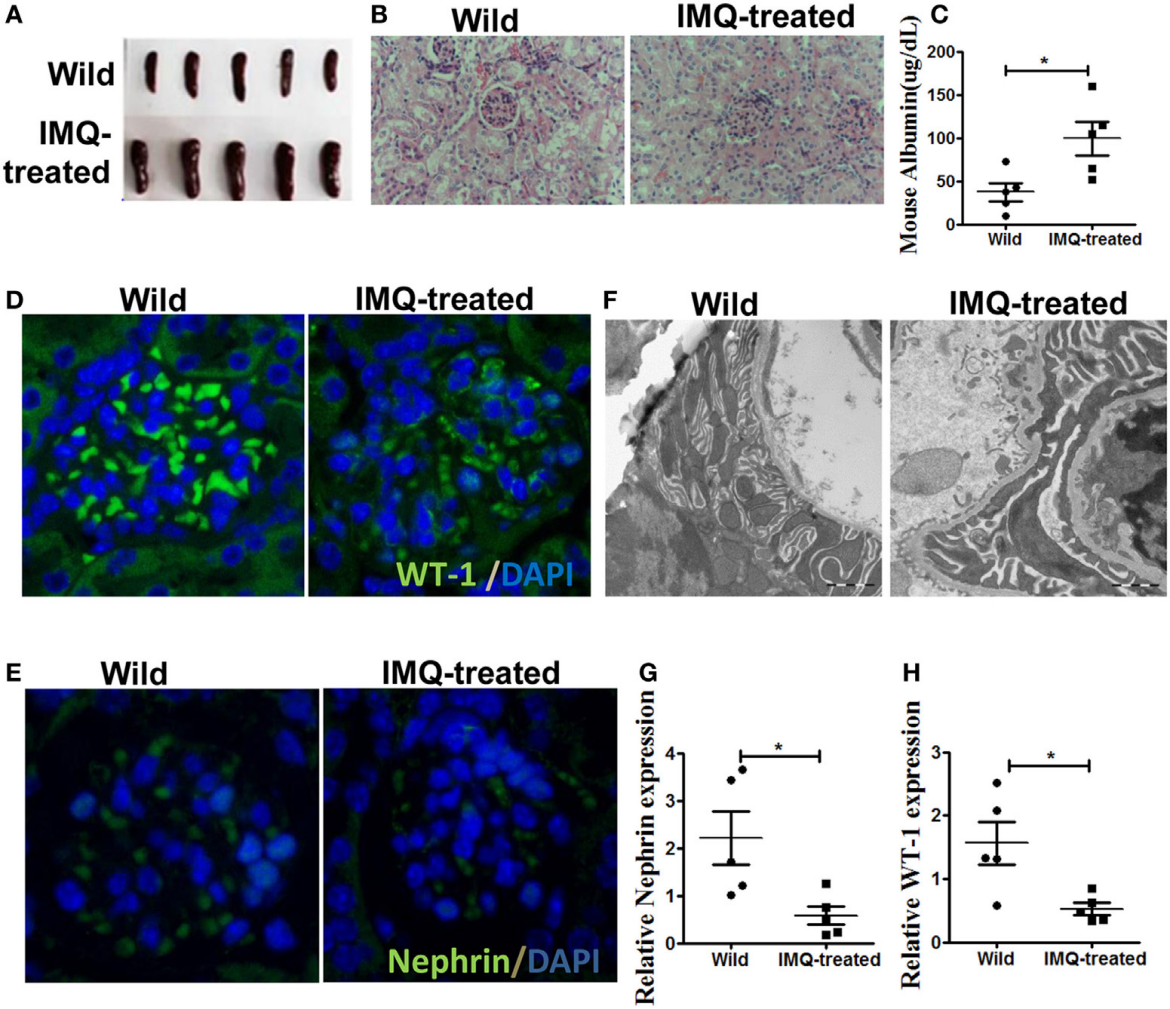
Podocyte injury in IMQ-induced lupus mice. C57BL/6 mice were treated with IMQ or PBS for 10 weeks, sacrificed to spleens and kidneys for analysis. **(A)** Marked splenomegaly in IMQ-treated mice, compared with wild mice. **(B)** PAS staining of kidneys histologic differences in wild mice and IMQ mice. **(C)** Proteinuria in wild mice and IMQ mice was determined using Mouse Albumin ELISA Quantitation Set. **(D,E)** Immunofluorescence of glomerular Wilms’ tumor protein (WT-1) **(D)** and nephrin **(E)**. **(F)** Assessment of podocyte foot processes by transmission electron microscopy. **(G,H)** Expression of nephrin and WT-1 in wild mice and IMQ-treated mice was determined by quantitative real-time RT-PCR. Data represent the mean scores ± SEM. *P ≤ 0.05, **P ≤ 0.01, ***P ≤ 0.001.

It has been report that TLR-7 is involved in the development of SLE (26). The up-regulation of TLR-7 in patient with SLE and TLR-7 activation in wild-type mice leads to lupus-like systemic autoimmune disease with elevated levels of autoantibodies and multiple organ involvement (27, 28). According to previous study (28), we initially applied imiquimod (IMQ), a TLR-7 agonist, to induce lupus mice. Consistent with previous report, the spleens of IMQ-treated mice became swollen (Figure 1A) and significant changes have been found in renal morphology (Figure 1B). Moreover, the level of urinary protein was significantly higher in urine of IMQ-treated mice than in normal controls (Figure 1C). Furthermore, WT-1 and nephrin are required during kidney development for the maturation of podocytes and their expressions are down-regulated in acquired glomerular diseases. The deficiency of WT-1 and nephrin is considered a pathologic feature of glomerular injury. The analysis of immunofluorescence showed that the expression of WT-1 and nephrin was obviously decreased in kidney of IMQ-treated mice compared with wild mice (Figures 1D,E). The down-regulated expression of WT-1 and nephrin at mRNA level was confirmed by qRT-PCR (Figures 1G,H). Meanwhile, electron microscopy revealed that the foot process effacement of podocyte was exacerbated in the IMQ-treated mice compared with wild mice. These results indicated that podocyte injury of the glomerulus was obviously more serious in TLR-7 agonist imiquimod-induced lupus mice than normal mice.

### Expansion of MDSCs Correlates Positively With Podocyte Injury in Imiquimod-Induced Lupus Mice

**Figure 2:**
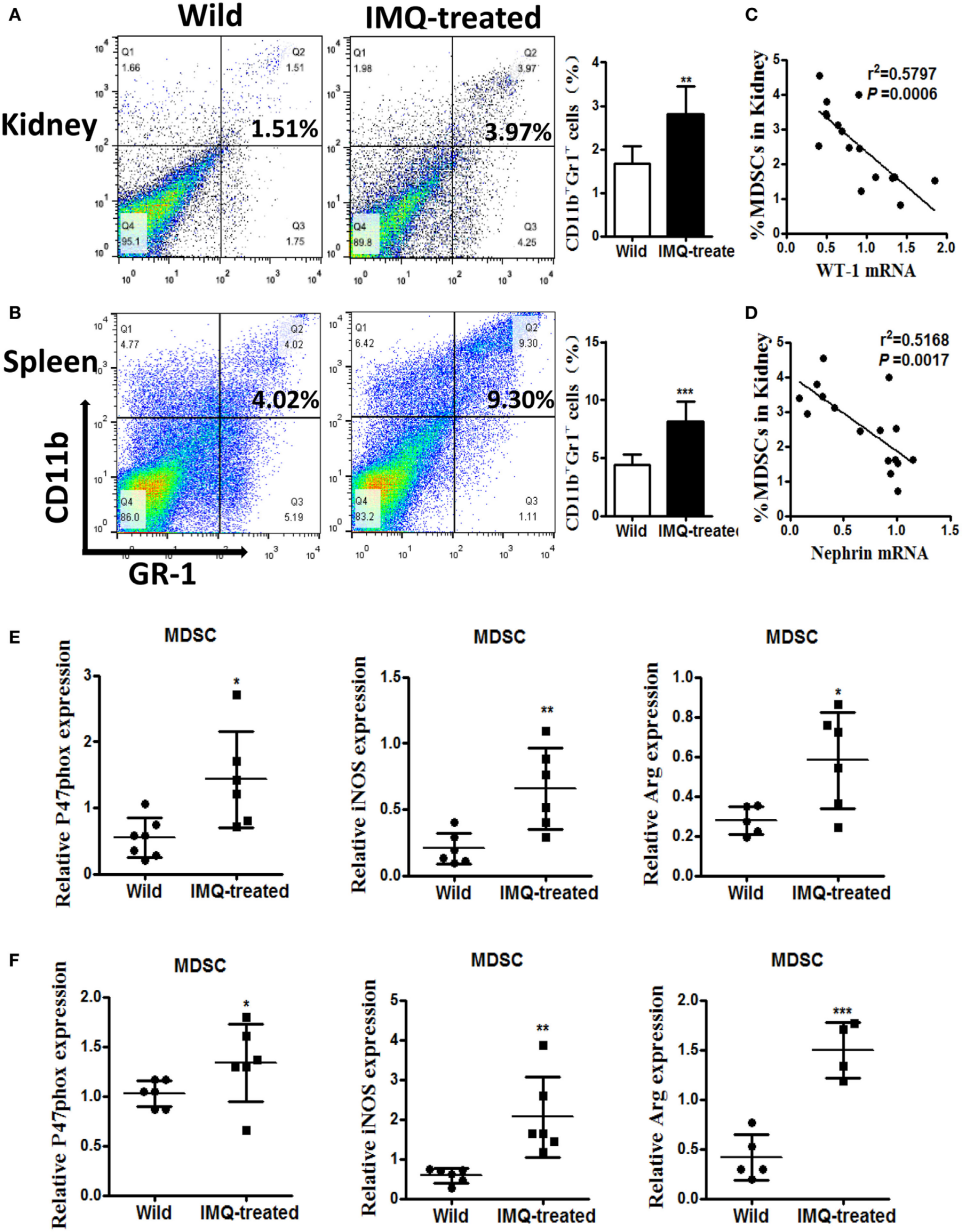
Expansion of myeloid-derived suppressor cells (MDSCs) in IMQ-treated mice. **(A,B)** The frequency of renal **(A)** and splenic **(B)** MDSCs in wild mice and IMQ-treated mice were determined by FACS, right panel is the statistics for the results of flow cytometer. **(C,D)** Correlation analysis between mRNA of Wilms’ tumor protein (WT-1) **(C)**, nephrin **(D)**, and the frequency of MDSCs in IMQ-treated mice. Each symbol represents an individual mouse (WT-1: *n* = 16, *r*^2^ = 0.5797, *P* = 0.0006; nephrin: *n* = 16, *r*^2^ = 0.5168, *P* = 0.0014). **(E)** Expression of P47phox, inducible nitric oxide synthase, and arginase-1 in MDSCs purified from kidneys of wild mice and IMQ-treated mice was measured by quantitative real-time RT-PCR (qRT-PCR). **(F)** Expression of functional molecules from splenic MDSCs of wild mice and IMQ-treated mice was determined by qRT-PCR. Data represent the mean scores ± SEM. *P ≤ 0.05, **P ≤ 0.01, ***P ≤ 0.001.

Previously, we found significant expansion of MDSCs in lupus mice, and expanded MDSCs correlated positively with disease severity in diseased lupus-prone mice (13). Here, we verified the percentage of MDSCs in kidneys and spleens by flow cytometry again, and the results showed the frequency of MDSCs markedly elevated in both kidneys and spleens of IMQ-treated mice compared with wild-type mice (Figures 2A,B). We further examined whether the expansion of MDSCs was associated with the decreased expression of WT-1 and nephrin. The results showed the expanded MDSCs were negatively correlated with the reduced expression of WT-1 and nephrin at mRNA level (Figures 2C,D). Expectedly, the function of MDSCs was also changed, i.e., the expression levels of their functional genes, including NOX component P47^phox^, iNOS, and Arg-1 were evidently increased in MDSCs purified from kidneys and spleens of IMQ-treated mice (Figures 1E,F and 2E,F). These results indicate the expanded MDSCs correlated positively with renal injury severity in the inducible lupus murine model and may be involved in podocyte injury of nephropathy damage process in LN.

### TLR-7-Activated MDSCs Induce Podocyte Injury *In Vitro*

**Figure 3:**
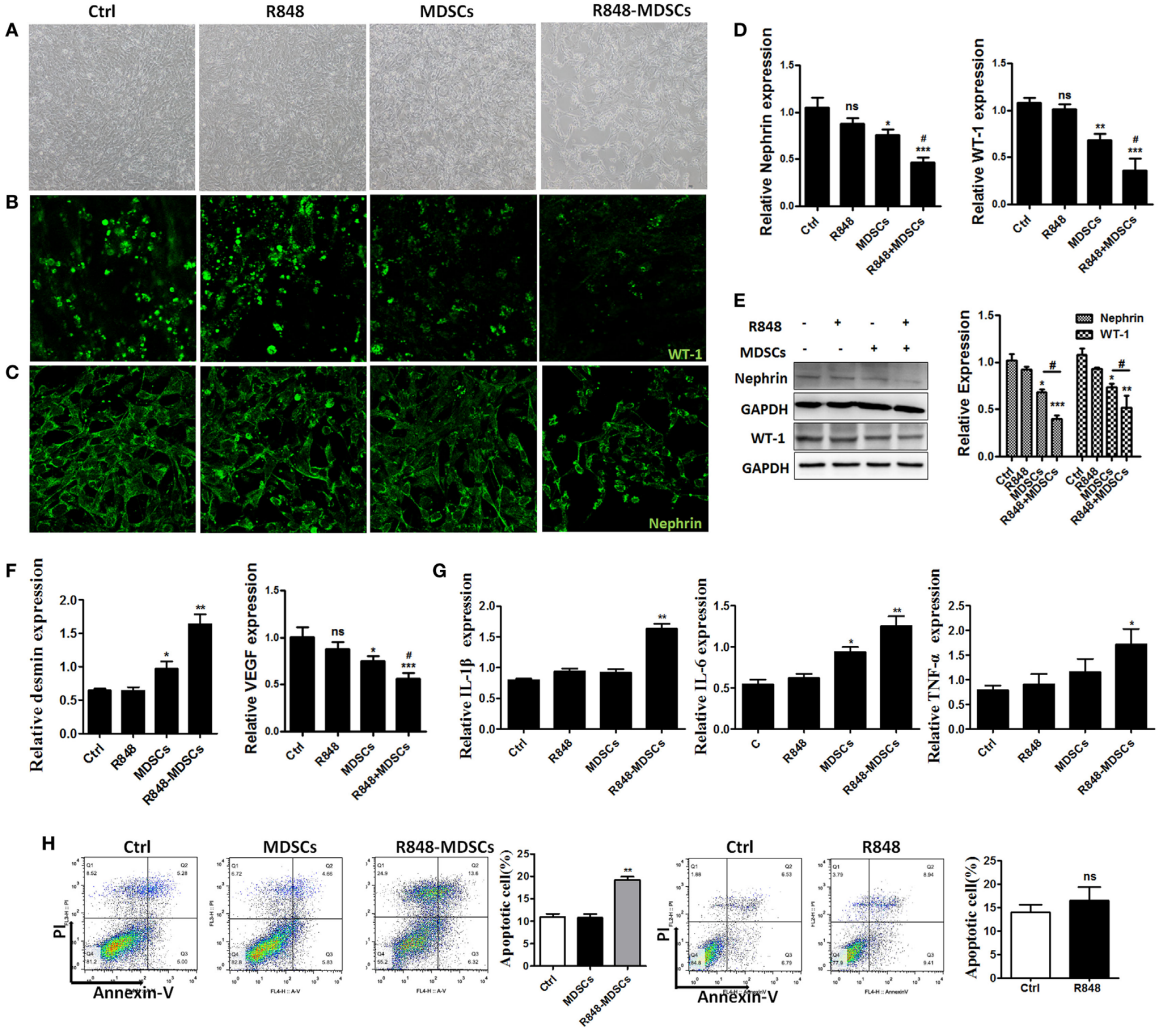
Toll-like receptor-7 (TLR-7)-activated myeloid-derived suppressor cells (MDSCs) induce podocyte injury. BM-derived MDSCs were co-cultured with mouse podocytes at ratio of 1:1 using transwell co-culture systems. **(A)** Representative light photomicrographs of podocytes after 48 h co-culture with MDSCs. **(B,C)** Expression of Wilms’ tumor protein (WT-1) **(B)** and nephrin **(C)** in podocytes was determined by immunofluorescence after 48 h co-culture with MDSCs. **(D,E)** Expression of nephrin and WT-1 in podocytes was determined by quantitative real-time RT-PCR (qRT-PCR) after 24 h co-culture with MDSCs **(D)** and Western Blot for nephrin and WT-1 after 48 h co-culture with MDSCs **(E)**. **(F)** Expression of desmin and vascular endothelial growth factor in podocytes was determined by qRT-PCR after 24 h co-culture with MDSCs. **(G)** Podocyte apoptosis was assessed with Annexin V and propidium iodide by flow cytometry after 48 h co-culture with MDSCs. **(H)** Expression of IL-1β, IL-6, and TNF-α in podocytes was measured by qRT-PCR after 24 h co-culture with MDSCs. Data represent the mean scores ± SEM. *P ≤ 0.05, **P ≤ 0.01, ***P ≤ 0.001.

To explore the specific role of MDSCs in podocyte injury, we respectively incubated podocytes alone as control group, treated podocytes with R848 as positive group, co-cultured podocytes with BM-induced MDSCs by transwells *in vitro*, stimulated with or without resiquimod (R848), a TLR-7 agonist. The results showed that most significant change was observed in the live podocytes co-cultured with R848-stimulated MDSCs, while morphologic change was also observed in co-cultured group of MDSCs. But no change was observed in control group and positive control group (Figure 3A). Moreover, the significant reduced expression of WT-1 and nephrin, two molecular markers of podocytes, was determined by immunofluorescence in the co-cultivation group of podocytes and R848-stimulated MDSCs (Figures 3B,C). In consistence with these results, the expression of WT-1 and nephrin was also manifestly decreased at both mRNA level and protein levels in the co-cultured group of podocytes and R848-treated MDSCs (Figures 3D,E). In addition, the expression of desmin, which is a sensitive marker of podocyte injury (29), was up-regulated at mRNA level in the co-cultured podocytes and R848-treated MDSCs, compared to control groups (Figure 3F). As previous study shown, podocytes can also secrete vascular endothelial growth factor (VEGF) to maintain the integrity of glomerular endothelial cells and promote the formation of new capillaries (17). The expression of VEGF was measured by qRT-PCR and the results showed that the down-regulated expression of VEGF in podocytes co-cultured with R848-treated MDSCs (Figure 3F). These data reveal that TLR-7-activated MDSCs indeed induce podocyte injury *in vitro*.

To further directly investigate the association of TLR-7-activated MDSCs with podocyte injury. We measured the cell apoptosis of podocytes alone, R848-treated, co-cultured with MDSCs stimulated with or without R848 using transwell by flow cytometer, respectively. The results showed that MDSCs treated with R848 promoted obviously podocytes apoptosis, compared with the treatment of podocytes alone (Figure 3**H**). Moreover, the expression of inflammatory factors, such as IL-1β, IL-6, and TNF-α were up-regulated markedly in podocytes co-cultured with MDSCs treated with R848 (Figure 3**G**). These results indicate TLR-7-activated MDSCs can cause podocyte apoptosis and induce podocytes to secrete inflammatory factors.

### Podocyte Injury Is Attributed to ROS From TLR-7-Activated MDSCs

**Figure 4:**
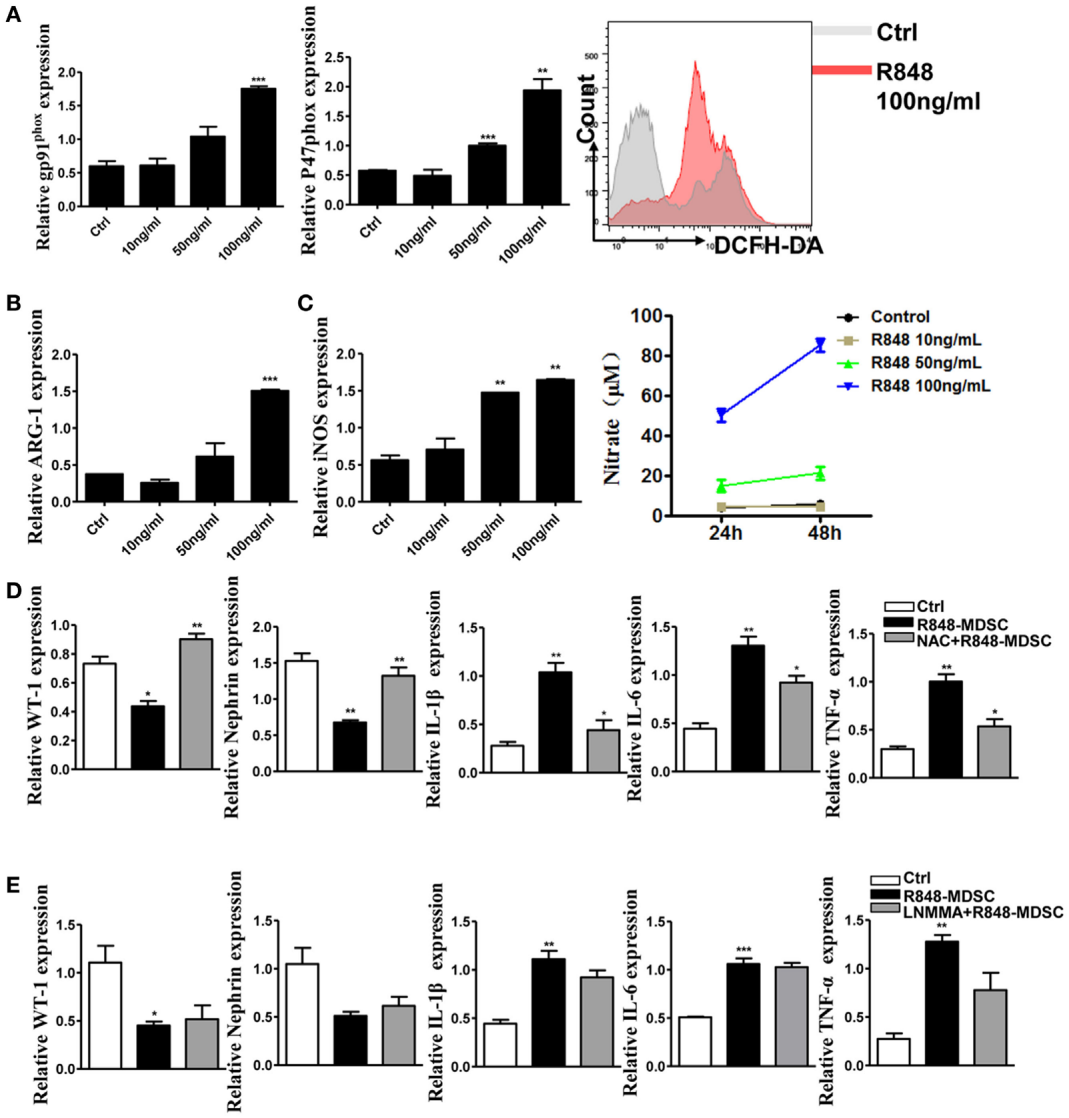
Toll-like receptor-7 (TLR-7) activation induces myeloid-derived suppressor cells (MDSCs) to generate reactive oxygen species (ROS). **(A)** Expression of gp91^phox^ and p47^phox^ in MDSCs was measured by quantitative real-time RT-PCR (qRT-PCR) after 12 h treatment with R848 (10, 50, and 100 ng/ml); MDSCs treated with R848 for 24 h, loaded with DCFDA. Fluorescence intensity of DCFDA was measured by FACS. **(B)** Expression of arginase-1 in MDSCs was measured by QPCR after 12 h treatment with R848. **(C)** Expression of inducible nitric oxide synthase (iNOS) in MDSCs was measured by qRT-PCR after 12 h treatment with R848; NO in the supernatant of MDSCs was determined using NO detection kit after 24 or 48 h treatment with R848. **(D)** Expression of Wilms’ tumor protein (WT-1), nephrin, IL-1β, IL-6, and TNF-α in podocytes was measured by qRT-PCR after 24 h co-culture with treated R848 with MDSCs pretreated with *N*-acetyl-l-cysteine (NAC) for 1 h. **(E)** Expression of WT-1, nephrin, IL-1β, IL-6, and TNF-α in podocytes was measured by qRT-PCR after 24 h co-culture with treated R848 with MDSCs pretreated with NAC for 1 h. BM-derived MDSCs were pretreated with ROS inhibitors *N*-acetyl-l-cysteine (NAC, 5 mM) or iNOS inhibitors L-NMMA (0.5 mM) for 1 h, and co-cultured with mouse podocytes at ratio of 1:1 using transwell co-culture systems. Data represent the mean scores ± SEM. *P ≤ 0.05, **P ≤ 0.01, ***P ≤ 0.001.

To elucidate the mechanism of TLR-7-activated MDSCs impair podocytes, we focused on functional molecules of MDSCs. The results showed that the functional molecules of MDSCs were significantly up-regulated in a dose-dependent effect of TLR-7 agonist R848 treatment (Figures 4A–C). The function of MDSCs was also enhanced by activating TLR-7 signaling. Previous study revealed that renal overexpression of ROS could directly lead to podocyte injury in diabetic nephritis model mice and increased production of oxidative stress markers in the renal tissue of SLE patients (30, 31). To further determine the role of the functional molecule of MDSCs in podocyte injury, we co-cultured podocytes with MDSCs treated with R848, or pretreated with ROS inhibitors *N*-acetyl-l-cysteine (NAC) or iNOS inhibitors L-NMMA for 1 h before treated with R848. The results showed that the expression of WT-1 and nephrin was increased, while the expression of IL-1β, IL-6, and TNF-α was reduced in podocytes co-cultured with MDSCs pretreated with NAC before treatment with R848, compared with control group, and co-cultured with MDSCs treated with R848 (Figure 4D). In neutralizing iNOS using L-NMMA, podocyte injury and the levels of inflammatory factors were not significantly alleviated (Figure 4E). The results indicated that podocyte injury may be attributed to ROS generation from TLR-7-activated MDSCs.

### ROS Promotes Podocyte Injury *via* p-38MAPK and NF-kB Signaling

**Figure 5:**
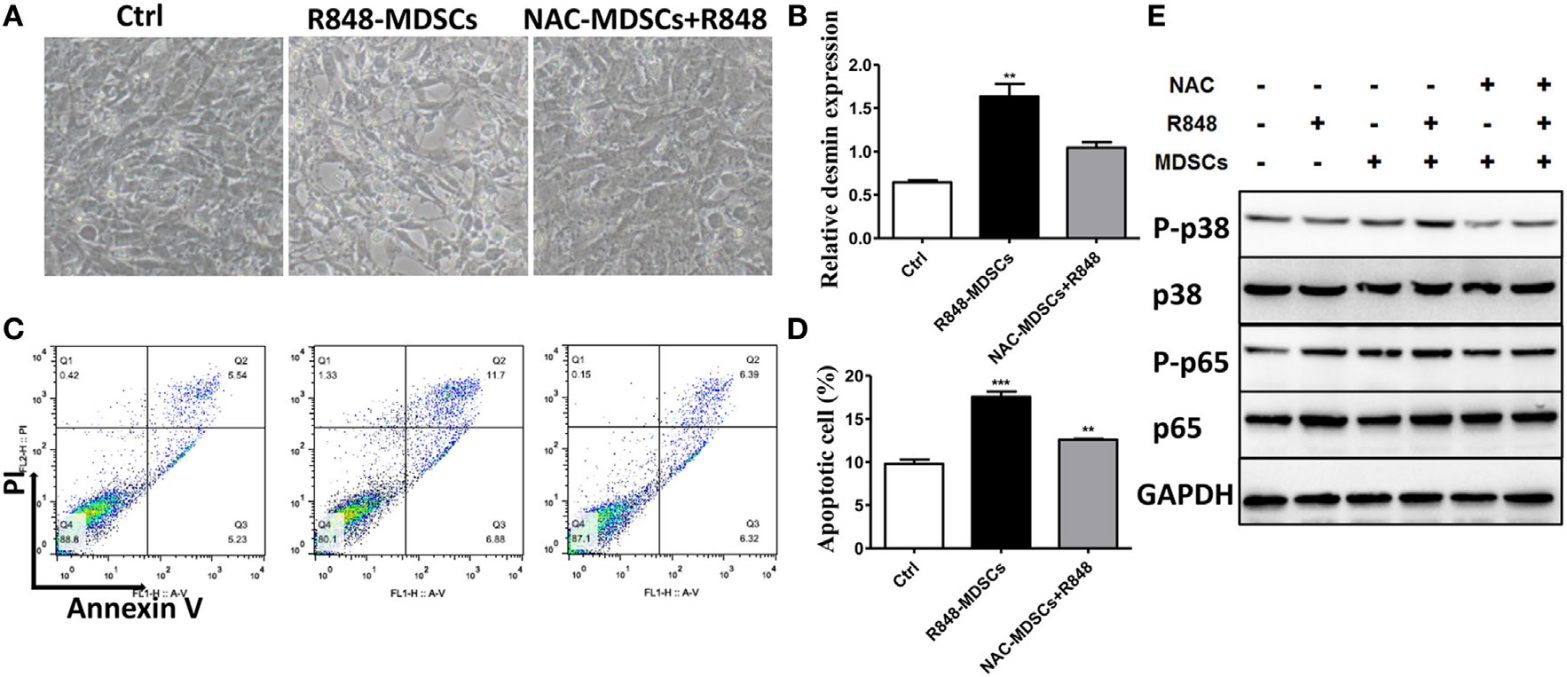
Myeloid-derived suppressor cells (MDSCs) induce podocyte injury by reactive oxygen species (ROS). BM-derived MDSCs were pretreated with ROS inhibitors *N*-acetyl-l-cysteine (5 mM) or inducible nitric oxide synthase inhibitors L-NMMA (0.5 mM) for 1 h, and co-cultured with mouse podocytes at ratio of 1:1 using transwell co-culture systems. **(A)** Representative light photomicrographs of podocytes after 48 h co-culture with MDSCs. **(B)** Expression of desmin in podocytes was measured by quantitative real-time RT-PCR after 24 h co-culture with MDSCs. **(C,D)** Podocyte apoptosis was assessed with annexin V by flow cytometry after 48 h co-culture with MDSCs **(C)** and the statistic results of podocyte apoptosis **(D)**. Data represent the mean scores ± SEM. **(E)** The protein levels of p-p38, p-38, p-p65, and p-65 were assessed by western blot and normalized to GAPDH. *P ≤ 0.05, ** *P* ≤ 0.01, ***P ≤ 0.001.

To verify further the effect of ROS secreted by MDSCs on podocyte injury, apoptosis of podocytes was tested. We first observed that podocytes became shrinking and round in cellular morphology when co-cultured with MDSCs treated with R848. In contrast, the change in podocytes was reversed when co-cultured by adding R848 to MDSCs after pretreated with NAC for 1 h (Figure 5A). Moreover, we detected podocyte injury marker desmin and cell apoptosis after co-incubated with R848-treated MDSCs following NAC-pretreatment. The results showed that NAC-pretreatment significantly reduced expression of desmin (Figure 5B). In consistence with the results, NAC-pretreatment obviously decreased the cell apoptosis of podocytes (Figures 5C,D). These results indicate that inhibition of ROS by NAC markedly blocked the deteriorative effect of MDSCs on podocyte injury. In addition, previous studies have shown that ROS might promote podocyte injury *via* activation of p-38MAPK or NF-kB pathway (32, 33). Thus, we measured the protein expression of p-p38MAPK, p-38MAPK, p-p65, and p-65 by Western blot. The results showed that the expressions of p-p38MAPK and p-p65 were significantly increased in podocytes co-cultured with R848-stimulated MDSCs, compared with those in other group of the treated podocytes (Figure 5F). These data indicate that TLR-7-activated MDSCs can induce podocyte injury through ROS-activated p-38MAPK and NF-kB pathways.

### Correlation of Podocyte Injury With Expansion of MDSCs Is Verified in Pristane-Induced Lupus Mice

**Figure 6:**
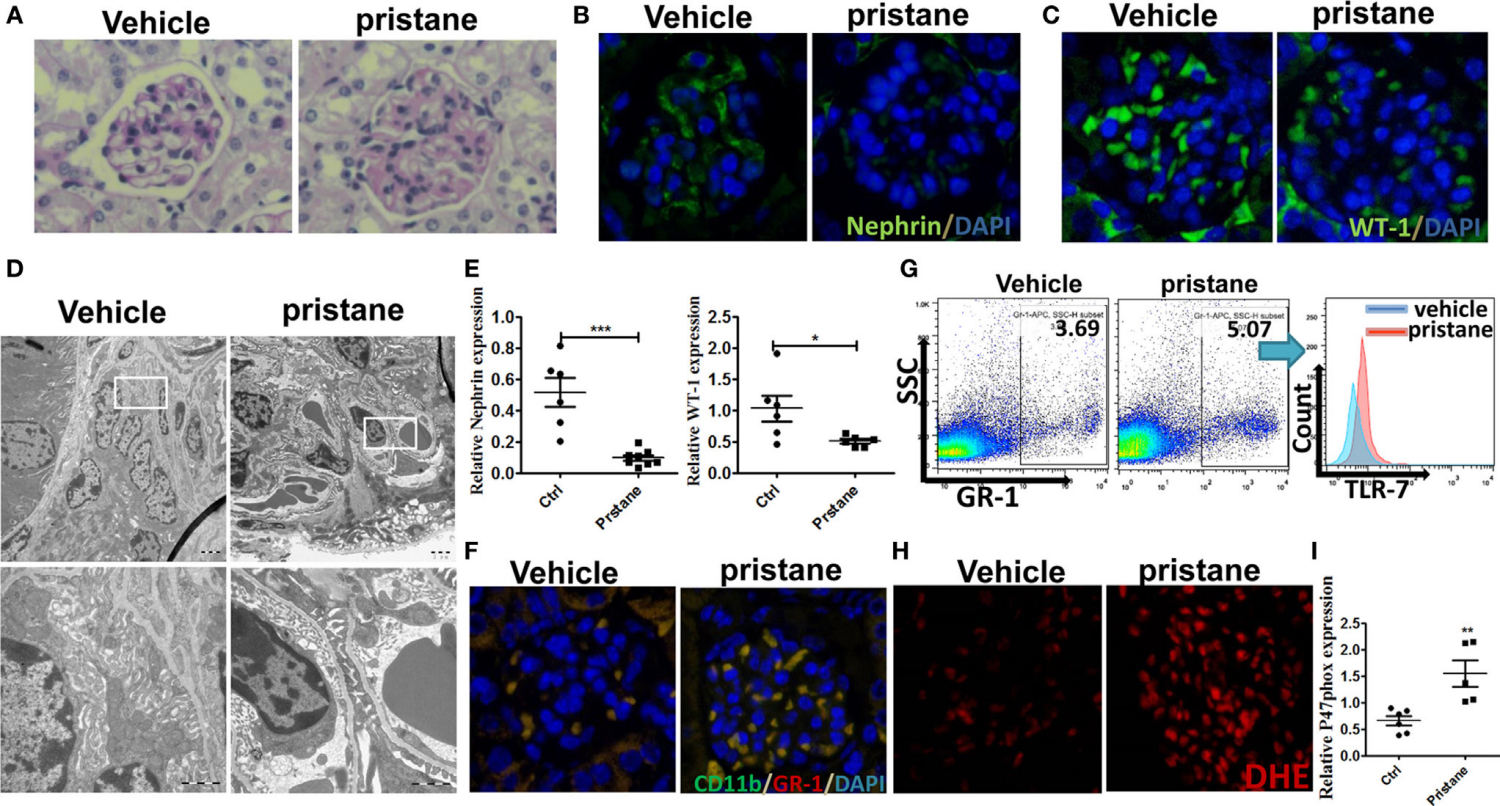
Podocyte injury and expansion of myeloid-derived suppressor cells (MDSCs) in pristane-induced lupus mice. **(A)** PAS staining in kidneys of mice treated with or without pristane for 7 months. **(B,C)** Immunofluorescence of glomerular nephrin **(B)** and WT-1 **(C)** in kidney samples from BALB/c mice treated with PBS or pristane. **(D)** Assessment of podocyte foot processes by transmission electron microscopy. Boxed area in the above panel is shown at higher magnification at below. Original magnification 6,000× (up) and 25,000 (down). **(E)** Expression of nephrin and WT-1 was determined by quantitative real-time RT-PCR (qRT-PCR) in kidney from PBS or pristine-treated BALB/c mice. **(F)** Immunofluorescence of glomerular CD11b and GR-1, represent MDSCs, in PBS or pristine-treated BALB/c mice. **(G)** Expression of toll-like receptor-7 in renal MDSCs of PBS or pristine-treated BALB/c mice was determined by FACS. **(H)** The differences of reactive oxygen species in glomerulus were detected by DHE staining between wild mice and pristane-treated mice. **(I)** Expression of P47^phox^ in MDSCs purified from kidneys of PBS or pristine-treated BALB/c mice was measured by qRT-PCR. Data represent the mean scores ± SEM. *P ≤ 0.05, **P ≤ 0.01, ***P ≤ 0.001.

Pristane, an isoprenoid alkane induces a lupus-like syndrome in several non-autoimmune prone mouse strains (34). We constructed the pristane-induced lupus-prone mice and confirmed that the model mice showed significant renal damage. Consistent with the results of IMQ-treated mice, 7 months after pristane or PBS challenge, histologic sections were stained by PAS and analyzed for pathologic features. The results showed that glomerular injury with basement membrane thickening, mesangial matrix expansion, endocapillary hypercellularity, and PAS positive deposits in pristane-induced lupus mice (Figure 6A). Immunofluorescence staining of renal tissues showed that the number of WT-1^+^ and nephrin^+^ podocytes was significantly lower in the glomerular of pristane-treated mice than in PBS-treated mice (Figures 6B,C). Moreover, the results of electron microscopy revealed the exacerbated podocyte foot process effacement in pristane-treated mice compared with wild mice (Figure 6D). We also found that the expression levels of WT-1 and nephrin were significantly down-regulated in the kidneys of pristane-treated mice by qRT-PCR (Figure 6E). The above results suggest that pristane-induced lupus model mice encounter obvious renal damage, especially the glomerular podocyte injury. Of note, percentage of MDSCs was also increased by immunofluorescence staining for CD11b and GR-1 in the kidneys of pristane-treated mice (Figure 6F). Moreover, TLR-7 amplified the activation of MDSCs (Figure 6G). The overexpression of ROS was also detected by ROS fluorescent probe, DHE, in the pristane-treated mice glomerular (Figure 6H). The mRNA level of P47phox was up-regulated in kidneys of pristane-treated mice (Figure 6I). These results verified that podocyte injury was correlated with expansion of MDSCs and their ROS secretion in LN of pristane-induced lupus mice.

## Discussion

Increasing studies suggest the expansion of MDSCs populations in murine models and patients with autoimmune diseases, especially SLE. These studies also indicate the association of MDSCs with the different degrees of LN in patient with SLE. A recent study demonstrated that expanded populations of both M-MDSCs and G-MDSCs and their frequency positively correlated with serum ARG1 concentration, Th17 cell responses and the associated renal injuries in patients with SLE and a SLE model (11). According to previous results, a significant increase in MDSCs was correlated positively with renal injury in LN. Previously, we have already demonstrated that the expansion of splenic MDSCs and the degrees of renal injuries was increased with disease progression in SLE (13). In this study, we further confirmed significant renal injury symptoms, proteinuria, and the expansion of MDSCs in spleens and kidneys in both pristane and imiquimod-induced lupus-like model mice. These results suggested that MDSCs might be involved in the immune regulation of renal microenvironment and induce kidney lesions.

Podocytes are highly differentiated cells characterized by expressing markers, such as synaptopodin, Wilms’ tumor protein (WT-1), glomerular epithelial protein 1, and nephrin. These molecules play important roles to maintain filtration barrier integrity (35). In this study, we found the decreasing expression of WT-1 and nephrin by immunofluorescence and qRT-PCR in kidneys of both pristane and imiquimod-induced lupus-like model mice. These results indicated severe podocytes injury. Furthermore, we found the frequency of MDSCs was negatively correlated with the expression of WT-1 and nephrin in pristane- or imiquimod-induced lupus-like model mice, which has been demonstrated the correlation with nephrotic-range proteinuria and extensive effacement of podocyte foot processes in LN (17, 36). *In vitro*, the co-cultivation experiment of MDSCs and podocytes shown MDSCs could lead to the down-regulated expression of podocytes markers, WT-1 and nephrin, at mRNA level and protein level detected by immunofluorescence and qRT-PCR. Moreover, the up-regulated expression of desmin and the down-regulated expression of VEGF were significant in co-cultivation experiments. The damage effect was enhanced by R848, and the results were further demonstrated by cell apoptosis assay. In addition, R848-treated MDSCs promoted the up-regulated expression of IL-1β, IL-6, and TNF-α in podocytes. A recent report demonstrated that IL-1β, IL-6, and TNF-α can influence the permeability of the glomerular basement membrane and alter glomerular filtration in a ROS-dependent mean (37). These data indicated that MDSCs could possibly affect podocytes injury with a ROS-dependent way to promote renal injuries in LN and the activation of TLR-7 in MDSCs exerted an important role.

It has been reported that endogenous agonists of TLR-4 were present in glomerular diseases that may induce podocyte dysfunction independent of TIL7/9 activation seen with endocytosed DNA and RNA immune complexes in SLE (38). Moreover, a number of studies demonstrated that LPS and oligosaccharide could cause comparable podocyte injury *in vitro* and *in vivo* (39). In the present study, TLR-7 activation of MDSCs occurred in kidneys of IMQ-treated mice and pristane-treated mice. Furthermore, ROS was increasing secreted by MDSCs stimulated with R848 and *in vitro* and *in vivo*. These results suggest that the role of TLR-7 activation is important in the generation of ROS of MDSCs. A common link of acute and chronic kidney injuries with the enhanced generation of ROS and reactive nitrogen species during injury progression was documented (40–42). Here, although the level of iNOS and NO was up-regulated in R848-treated MDSCs, the expression of WT-1 and nephrin was not significantly reversed in podocytes co-cultured with L-NMMA-pretreated MDSCs treated with R848, compared with podocytes co-cultured with R848-treated MDSCs. These data suggested that TLR-7-activated MDSCs promote podocyte injury mainly by the increased generation of ROS.

In the present study, we demonstrated that MDSCs induce podocytes injury by ROS and were involved in the subsequent development of proteinuria in LN for the first time. Furthermore, TLR-7-activated MDSCs enhanced podocyte injury through activating p-38MAPK and NF-kB pathways by ROS. In summary, our study further defined the role of MDSCs in the pathogenesis of LN, and we proposed that therapeutic approaches directed against MDSCs may lead to alleviation of the disease. In a word, our findings deepen our understanding of the occurrence of LN and provide a new ideas and targets for the clinical treatment of LN.

## Ethics Statement

All aspects of this study were approved by the Research Ethics Committee of Nanjing University, and all experiments were conducted in accordance with institutional guidelines for animal care and used based on the Guide for the Animal Care Committee at Nanjing University. All these specimens were handled and anonymized according to ethical and legal standards.

## Author Contributions

DZ and JX conceived, designed the experiments, drafted the article, performed experiments, data analysis, and wrote the manuscript. JR, LD, GS, and DL supported the materials. YH provided the concept and designed this study. HD and YH co-designed experiments and co-wrote the manuscript. All authors reviewed and approved the manuscript final version.

## Conflict of Interest Statement

The authors declare that the research was conducted in the absence of any commercial or financial relationships that could be construed as a potential conflict of interest.
