# Supplementary material for: Myeloid-Derived Suppressor Cells Induce Podocyte Injury Through Increasing Reactive Oxygen Species in Lupus Nephritis
Source: Front Immunol. 2018 Jun 25;9:1443. doi: 10.3389/fimmu.2018.01443 (PMC6026681; doi:10.3389/fimmu.2018.01443)
Supplement: Supplementary file 1 [file Data_Sheet_1.docx]

**MDSCs Induce Podocyte Injury through Increasing Reactive Oxygen Species in Lupus Nephritis**

Dongya Zhang^1,#^, Jingjing Xu^1,#^, Jing Ren^1^, Liang Ding^1^, Guoping Shi^1^, Dan Li^1^, Huan Dou^1,2*^, Yayi Hou^1,2*^

**^1^**The State Key Laboratory of Pharmaceutical Biotechnology, Division of Immunology, Medical School, Nanjing University, Nanjing 210093, China.

**^2^** Jiangsu Key laboratory of Molecular Medicine, Nanjing 210093, China

**#** Contribute equally to this work

***Corresponding author**: Yayi Hou, The State Key Laboratory of Pharmaceutical Biotechnology, Division of Immunology, Medical School, Nanjing University, No.22 Hankou Rd., Gulou District, Nanjing 210093, Jiangsu, China. Tel/Fax: +86-25-83686341; Email: yayihou@nju.edu.cn

Or Huan Dou, The State Key Laboratory of Pharmaceutical Biotechnology, Division of Immunology, Medical School, Nanjing University, No.22 Hankou Rd., Gulou District, Nanjing 210093, Jiangsu, China. Tel/Fax: +86-25-83686441; E-mail: douhuan@nju.edu.cn

**Supplementary Table S1**

Table S1. Primers of mouse gene used for real-time RT-PCR

| **Gene** | **Forward primer (5’-3’)** | **Reverse primer (5’-3’)** |
| --- | --- | --- |
| Nephrin | ATGGGAGCTAAGGAAGCCACA | CCACACCACAGCTTAACTGTC |
| WT-1 | AGCACGGTCACTTTCGACG | GTTTGAAGGAATGGTTGGGGAA |
| p47phox | AGAACAGAGTCATCCCACAC | GCTACGTTATTCTTGCCATC |
| gp91phox | TCACATCCTCTACCAAAACC | CCTTTATTTTTCCCCATTCT |
| iNOS | CCAAGCCCTCACCTACTTCC | CTCTGAGGGCTGACACAAGG |
| ARG-1 | CTCCAAGCCAAAGTCCTTAGAG | GGAGCTGTCATTAGGGACATCA |
| desmin | CCTGGAGCGCAGAATCGAAT | TGAGTCAAGTCTGAAACCTTGGA |
| VEGF | CAATGATGAAGCCCTGGAG | TCTCCTATGTGCTGGCTTTG |
| IL-1β | GAAATGCCACCTTTTGACAGTG | TGGATGCTCTCATCAGGACAG |
| Il-6 | CTGCAAGAGACTTCCATCCAG | AGTGGTATAGACAGGTCTGTTGG |
| TNF-a | CAGGCGGTGCCTATGTCTC | CGATCACCCCGAAGTTCAGTAG |
| GAPDH | AGGTCGGTGTGAACGGATTTG | GGGGTCGTTGATGGCAACA |

**Supplementary Figure 1**

**
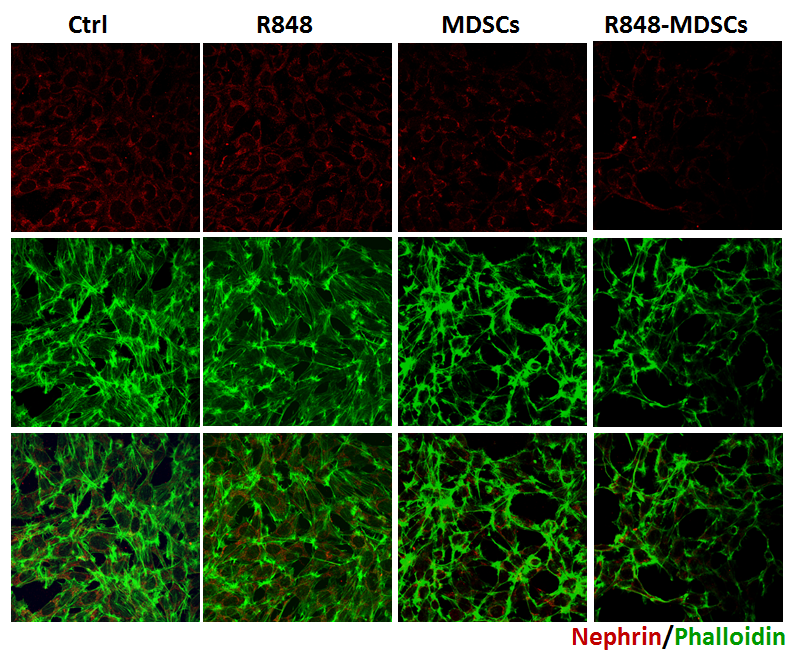
**

**Supplementary Figure 1.** TLR-7-activated MDSCs induce podocyte injury. BM-derived MDSCs were co-cultured with mouse podocytes at ratios of 1:1 using transwell co-culture systems. Expression of Nephrin in podocytes was determined by immunofluorescence after 48 hours co-culture with MDSCs. Cytoskeleton marked by Alexa fluro 488-Phalloidin was determined by immunofluorescence after 48 hours co-culture with MDSCs.

**Supplementary Figure 2**

**
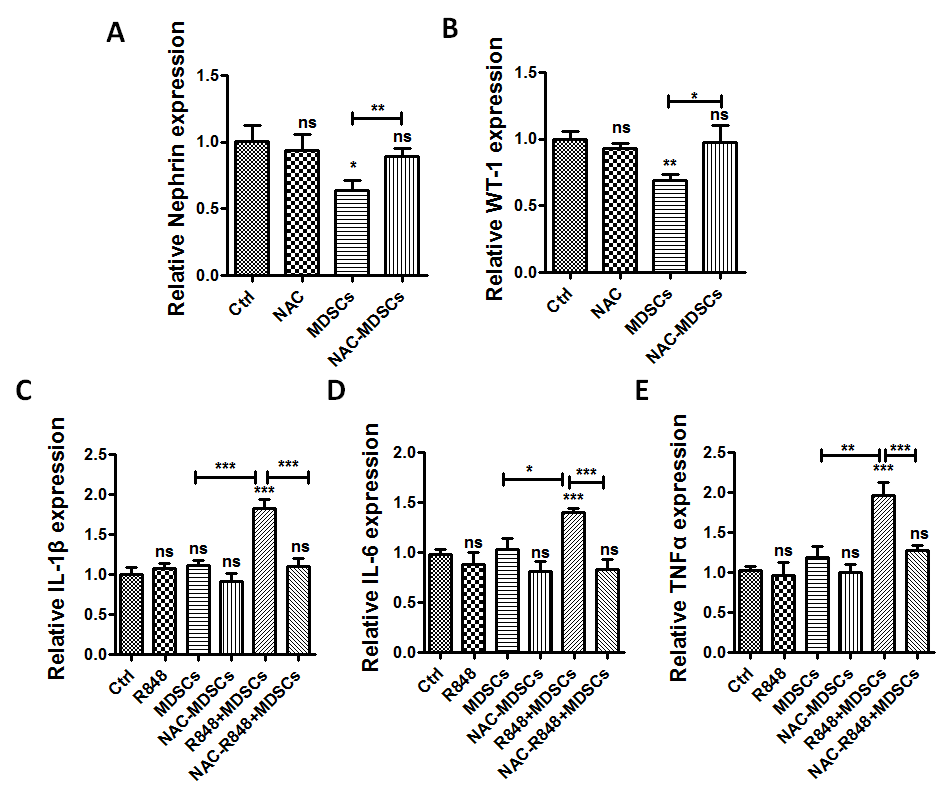
**

**Supplementary Figure 2.** TLR-7 activation promotes generation of ROS by MDSCs. **(A,B)** Expression of Nephrin and WT-1 in podocytes was determined by qRT-PCR after 24 hours co-culture with MDSCs with N-acetyl-L-cysteine (NAC, 5mM) pretreatment for 1h. **(C, D, E)** Expression of IL-1β, IL-6, and TNF-α in podocytes was measured by qRT-PCR after 24 hours co-culture with treated R848 with MDSCs pretreated with NAC for 1h. Data represent the mean scores±SEM. (** P≤0.05, ** P ≤0.01, *** P ≤0.001*).

**Supplementary Figure 3**

**
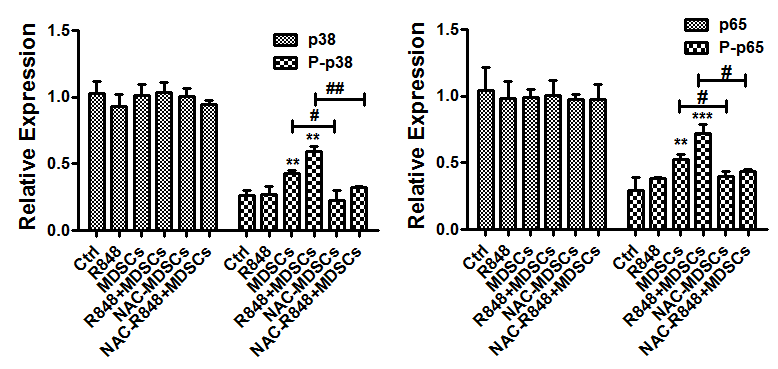
**

**Supplementary Figure 3.** MDSCs induce podocyte injury by ROS. The relative expression of p38, P-p38, p65 and P-p65 to GAPDH was calculated. (**,* **#** *P≤0.05, **,***##** *P ≤0.01, ***,***###** *P ≤0.001*).
